# Synthesis, Radiolabeling, and Biodistribution Study of a Novel DOTA-Peptide for Targeting Vascular Endothelial Growth Factor Receptors in the Molecular Imaging of Breast Cancer

**DOI:** 10.3390/pharmaceutics16070899

**Published:** 2024-07-04

**Authors:** Fatemeh Ebrahimi, Nooshin Reisi Zargari, Mehdi Akhlaghi, S. Mohsen Asghari, Khosrou Abdi, Saeed Balalaie, Mahboobeh Asadi, Davood Beiki

**Affiliations:** 1 Department of Nuclear Pharmacy, School of Pharmacy, Tehran University of Medical Sciences, Tehran 1417614411, Iran; 2 University Campus II, University of Guilan, Rasht 4144784475, Iran; nooshinzargar@webmail.guilan.ac.ir; 3 Research Center for Nuclear Medicine, Shariati Hospital, Tehran University of Medical Sciences, Tehran 1411713135, Iran; m-asadi@farabi.tums.ac.ir; 4 Institute of Biochemistry and Biophysics (IBB), University of Tehran, Tehran 1417614335, Iran; sm.asghari@ut.ac.ir; 5 Peptide Chemistry Research Institute, K. N. Toosi University of Technology, Tehran 158754416, Iran

**Keywords:** Gallium-68, vascular endothelial growth factor receptor, peptide, PET imaging, breast cancer

## Abstract

As angiogenesis plays a pivotal role in tumor progression and metastasis, leading to more cancer-related deaths, the angiogenic process can be considered as a target for diagnostic and therapeutic applications. The vascular endothelial growth factor receptor-1 (VEGR-1) and VEGFR-2 have high expression on breast cancer cells and contribute to angiogenesis and tumor development. Thus, early diagnosis through VEGFR-1/2 detection is an excellent strategy that can significantly increase a patient’s chance of survival. In this study, the VEGFR1/2-targeting peptide VGB3 was conjugated with 1,4,7,10-tetraazacyclododecane-1,4,7,10-tetraacetic acid (DOTA), using 6-aminohexanoic acid (Ahx) as a spacer to prevent steric hindrance in binding. DOTA-Ahx-VGB3 was radiolabeled with Gallium-68 (^68^Ga) efficiently. An in vitro cell binding assay was assessed in the 4T1 cell line. The tumor-targeting potential of [^68^Ga]Ga-DOTA-Ahx-VGB3 was conducted for 4T1 tumor-bearing mice. Consequently, high radiochemical purity [^68^Ga]Ga-DOTA-Ahx-VGB3 (RCP = 98%) was prepared and stabilized in different buffer systems. Approximately 17% of the radiopeptide was internalized after 2 h incubation and receptor binding as characterized by the IC_50_ value being about 867 nM. The biodistribution and PET/CT studies revealed that [^68^Ga]Ga-DOTA-Ahx-VGB3 reached the tumor site and was excreted rapidly by the renal system. These features convey [^68^Ga]Ga-DOTA-Ahx-VGB3 as a suitable agent for the noninvasive visualization of VEGFR-1/2 expression.

## 1. Introduction

Globally, breast cancer (BC) is known as the most common type of cancer among females. In 2020, approximately 2.3 million new breast cancer cases were observed, and about 685,000 deaths occurred, representing one in every six cancer deaths in women. It has been predicted that by 2040, the incidence of breast cancer will increase to over 3 million new cases and about 1 million deaths worldwide per year (growing by over 50%). It is well established that focusing on early diagnosis through imaging modalities and increasing awareness, in addition to improving treatment, can reduce breast cancer mortality [1,2].

Metastasis is also the major cause of poor prognosis and cancer-related deaths, in which tumor angiogenesis plays a pivotal role. Among various angiogenic factors, vascular endothelial growth factors (VEGFs) and their receptors are essential mediators, having key roles in regulating endothelial cell (EC) proliferation and microvascular permeability. VEGF-A is one of the principal members of the VEGF family that can activate two vascular endothelial growth factor receptors (VEGFRs), VEGFR-1 and VEGFR-2 [3,4]. These receptors typically consist of an extracellular-ligand-binding domain (ECD) with a seven immunoglobulin (Ig)-like motif, a single transmembrane domain, a juxtamembrane domain, and a split tyrosine-kinase domain [5]. Considerably, VEGFR-1/2 has high expression in diverse cancers such as glioblastoma [6], lung cancer [7], pancreatic cancer [8], colorectal cancer [9], hepatocellular carcinoma [10], and leukemia [11]. In particular, many studies have also focused on the high-level expression of both receptors on breast cancer cells and their application as a prognostic marker [12,13,14,15,16]. Thus, early diagnosis through VEGFR-1/2 detection is an excellent strategy that can significantly improve outcomes for all breast cancer patients. Several types of molecules have been utilized for specific targeting of the VEGF/VEGFR axis, including VEGF and its derivatives, monoclonal antibodies (mAb) against VEGF or VEGFR (e.g., bevacizumab, ranibizumab) [17], VEGFR-targeting peptides and proteins [18], and tyrosine kinase inhibitors (TKIs) (e.g., imatinib, sunitinib, and sorafenib) [19,20]. Currently, more than ten TKIs have been approved for clinical use, in which the earlier generation demonstrated poor kinase specificity, while recent inhibitors more specifically target VEGFR [20,21]. In addition, the other successful agents applied in cancer targeting include anti-VEGF mAb bevacizumab (Avastin); the Fab fragment ranibizumab (Lucentis), approved in 2004 and 2006, respectively; and the humanized rabbit anti-VEGF monoclonal antibody BD0801 in phase III clinical developmental stage. However, although neutralizing antibodies such as ramucirumab (humanized anti-VEGFR2 mAb) show high specificity and long half-life, they have some limitations due to immunogenicity and high production costs [21,22].

Among many of the tumor-targeting ligands, peptides have some superior advantages compared to other molecules such as monoclonal antibodies, including better intratumoral diffusion, non-immunogenicity, lack of toxicity, specificity, high potency, low molecular weight, and easy and inexpensive synthesis. Hence, peptide ligands can be a favorable choice for drug delivery as diagnostic and therapeutic agents [23,24,25]. These targeting peptides can be conjugated to the appropriate imaging moieties or radiolabeled with the help of various radiolabeling techniques to interact with specific receptors and provide two-dimensional (2D) or three-dimensional (3D) images, corresponding to the biodistribution of these radiotracers [26]. Over the past decades, a variety of peptides targeting the VEGF/VEGFR axis have been labeled with various radionuclides, including Technetium-99m [27,28], Rhenium-188 [29], Copper-64 [30], Lutetium-177 [31], and Gallium-68 [32]. For example, VEGF125−136, a 12-amino-acid peptide, was labeled with Rhenium-188 and Copper-64 for single-photon emission computed tomography (SPECT) and positron emission tomography (PET) imaging, respectively, demonstrating binding potency toward VEGFR-2 [29,30]. Also, Barta et al. developed two [^68^Ga]Ga-labeled peptides as new PET tracers for tumor angiogenic process imaging, through VEGFRs targeting [33]. Gallium-68 can be an ideal diagnostic positron emission tomography (PET) isotope with proper physical properties and easy accessibility via a Germanium-68/Gallium-68 generator. This radiometal forms a thermodynamically stable complex by macrocyclic chelators such as 2,2′,2″,2‴-(1,4,7,10-tetraazacyclododecane-1,4,7,10-tetrayl)tetraacetic acid (DOTA) that can be conjugated directly or indirectly to the peptide [34]. DOTA and its derivatives are often used as accessible chelators for ^68^Ga radiolabelling. Additionally, two of the four Food and Drug Administration (FDA)-approved ^68^Ga radiopharmaceuticals are based on the DOTA macrocycle [35].

Therefore, according to our previous study that has demonstrated the anti-angiogenetic effect of a novel VEGFR1/2-targeting peptide with the sequence of NH2-Glu-Cys-Arg-Pro-Pro-Asp-Asp-Gly-Leu-Cys-COOH(VGB3) [36], this structure has been selected as a main compound for diagnostic purpose. VGB3 could interfere with VEGF binding to VEGFR-1/2 and inhibit angiogenesis and tumor growth via downregulation of the PI3K/AKT/mTOR and PLC_ϒ_/ERK1/2 pathways in human umbilical vein endothelial cells (HUVECs) and 4T1 cells, as well as inducing apoptosis through both intrinsic and extrinsic pathways [37]. Thus, the present study describes the peptide design, synthesis, radiolabeling, quality control, and biodistribution study of a [^68^Ga]Ga-DOTA-VGB3 as a diagnostic agent in healthy and tumor-bearing mice. To prevent steric hindrance in binding, using a proper spacer can extend binding affinity to the cognate receptor by increasing the distance between the peptide core and the DOTA chelator [38,39,40]. Thus, further modification was performed by adding a 6-aminohexanoic acid (Ahx) linker, as a common spacer that was used for earlier radiopharmaceuticals such as [^68^Ga]Ga-PSMA-11, to improve the binding affinity and tumor targeting [41,42].

## 2. Materials and Methods

### 2.1. Materials

Gallium-68 was eluted from a ^68^Ge/^68^Ga Generator System (30 mCi/day activity, Pars Isotope Co., Karaj, Iran) with 0.1 M HCl. Ultrapure water was purchased from Sigma Aldrich (Darmstadt, Germany) and used to prepare all aqueous solutions and buffers. Hydrochloric acid (Merck, Darmstadt, Germany) and normal saline were used for radiolabeling without further purification. Instant thin-layer chromatography (ITLC) was performed by a radio-TLC-scanner (RAYTEST, Straubenhardt, Germany) and glass fiber impregnated with silica gel (ITLC-SG, Merck, Darmstadt, Germany). Reverse-phase liquid chromatography (RP-HPLC) was performed for radiochemical purity assessment of the final product using an Agilent 1200 reverse-phase HPLC system equipped with a gamma detector (RAYTEST, Straubenhardt, Germany) and the mobile phase of water-trifluoroacetic acid 1% (*V*/*V*) and acetonitrile-trifluoroacetic acid 1%(*V*/*V*) (Aldrich Chemical Co., Darmstadt, Germany). pH was measured by pH indicator strips (Panpeha, Aldrich Chemical Co., Darmstadt, Germany). A NaI (Tl) gamma detector was applied for radionuclide purity determination and radioactivity measurement during animal biodistribution studies. The breast cancer cell line (4T1) was from the Pasteur Institute of Iran. Animal studies were carried out under the control of the Biomedical Research Ethics Committee of Tehran University of Medical Sciences (Ethical code: IR.TUMS.MEDICINE.REC.1400.501, 28 July 2021). All PET images were taken by a PET/CT imaging system (Biograph 6, Siemens Medical Solutions, Erlangen, Germany).

### 2.2. Synthesis of DOTA-Ahx-VGB3 and ^68^Ga-Radiolabeling

Peptide synthesis was performed using the protocol of solid-phase peptide synthesis (SPPS) developed by Merrifield [43]. This technique is based on a serial reaction of protected amino acids, in which the carboxyl group is activated to form an active ester by ammonium-derived coupling reagents and reacts with the N-terminus of the previous amino acid to form a peptide bond [44]. The obtained peptide was purified by preparative HPLC and lyophilized to prepare a white solid. Finally, the chemical structure and purity of the peptide were analyzed using mass spectrometry and HPLC.

Gallium-68, eluted in 3 mL of 0.1 M HCl from a ^68^Ge/^68^Ga-sterile generator, was added to the vial containing 320 μL sodium acetate buffer (1M, pH = 7) and peptide (25 μg). The reaction mixture was incubated at 95–105 °C for 10–12 min (pH = 2.5–3). The prepared product was cooled down and then loaded on the Sep-Pak C_18_ cartridge (Waters, Milford, MA, USA). Subsequently, the column was washed with normal saline to remove impurities. The final product was extracted with a mixture of ethanol and water (1:1) and filtered through a 0.22-micron filter (Merck Millipore, Darmstadt, Germany) into a sterilized vial [45]. The labeled molecule was diluted in normal saline to obtain an appropriate ethanol concentration for injection [46,47].

### 2.3. Quality Control

The radiochemical purity (RCP) was determined via ITLC and RP-HPLC. HPLC was performed on an Agilent 1200 system equipped with a NaI (Tl) gamma detector (Raytest, Straubenhrdt, Germany), using the gradient mixture of water (added 0.1% trifluoroacetic acid (TFA)) and acetonitrile (ACN, added 0.1% TFA): t: 0–3 min 0% ACN, 3–6 min 0–50% ACN, 6–15 min 50% ACN, 15–16 min 50–0% ACN, 16–20 min 0% ACN. The flow rate was set at 1 mL/min, for 20 min, using C_18_ column 150 × 3 mm, 3 μm (Thermo Fisher Scientific, Dreieich, Germany) [48].

ITLC was carried out on silica gel as the stationary phase and 1 M ammonium acetate/methanol mixture (1:1) as the mobile phase. The ITLC-SG strip analysis was then performed by a radio-TLC scanner.

### 2.4. Determination of n-Octanol/Water Partition Coefficient (Log p)

A total of 100 μL of the radiolabeled peptide was aliquoted into vials containing 0.99 mL of water and 1 mL of n-octanol, which was then mixed by a vortex mixer (Mabnagene, Tehran, Iran) at room temperature for 5 min. Aqueous and organic layers were separated by centrifugation (10 min, 5000 rpm). Then, 500 μL aliquots of each layer were transferred to the new vials, and the radioactivity of both was measured in a dose calibrator (Capintec CRC^®^-25, Florham Park, NJ, USA) [49].

### 2.5. In Vitro Stability of Radiopeptide

The stability of the radiotracer was determined by incubating the radiopeptide in various solutions and media, including normal saline (pH 5.5), human serum albumin, and 1M sodium acetate (pH 7). The diluted solutions were incubated at 37 °C with mild shaking. Samples of the incubation mixtures were spotted onto TLC at different time points (0, 15, 30, 60, 90, 120 min) and examined by a radio-TLC scanner. The radiochemical purity at different time intervals represented the stability of the [^68^Ga]Ga-DOTA-Ahx-peptide [50].

### 2.6. Radionuclide Identification and Activity Measurements

Radionuclide purity was assessed by gamma spectrometry and determination of the half-life of the radionuclide. Gallium-68 was eluted from the generator freshly and a dose calibrator measured the radioactivity of elution and the radiolabeled peptide. Then, the gamma spectrometry was performed based on the identification of the principal ϒ-photon (511 ± 20 KeV), using a gamma counter [51]. Half-lives were calculated by radioactivity measurement at different time intervals (0, 10, 15, 20 min) via dose calibrator and then put in the equation of t _½_ = 0.693 t/(ln A_0_/A_t_), wherein A_0_ = initial radioactivity, A_t_ = remaining radioactivity, and t = time [52].

### 2.7. Cell Culture and Animal Models

The 4T1 cell line was acquired from the Pasteur Institute of Iran (4T1 is a breast cancer cell line derived from the mammary gland tissue of a mouse BALB/c. (NCBICode: C604/the Pasteur Institute/Tehran/Iran). 4T1 cells are epithelial and can overexpress VEGFRs). The cells were cultured at 37 °C with 5% CO_2_ in Dulbecco’s modified Eagle’s medium (Gibco, Life Technologies, Carlsbad, CA, USA) supplemented with 10% fetal bovine serum (Gibco) and 1% antibiotics (penicillin and streptomycin) [53]. To develop tumor-bearing mice models, Balb/C mice (female, 6–8-week-old, 20–25 g) were purchased from the Institute of Biochemistry and Biophysics (IBB) (University of Tehran, Iran) and kept under the controlled conditions of temperature, humidity, and light/dark cycles with appropriate food and water (approved by the ethics committee of TUMS). Initially, the cells were harvested from the culture flask surface by 10 mM EDTA and centrifuged. After resuspending in serum-free culture medium, 1 × 10^6^ cells/100 μL were injected subcutaneously into the right shoulder of mice. In the next 10 days, the tumor size was ready to use for imaging and biodistribution studies with a volume of 5–8 mm^3^ [54].

### 2.8. Internalization and Surface Binding in the Breast Cancer Cell Line

A total of 1 × 10^6^ mammary carcinoma cells (NCBICode: C604/the Pasteur Institute/Tehran/Iran) were seeded per well into 12-well plates overnight at 37 °C in a cell culture incubator. On the day of the experiment, the 4T1 cells were treated with about 37 kBq (1 μCi) of [^68^Ga]Ga-DOTA-Ahx-peptide for different time intervals of 15, 30, 60, 120, and 240 min. Primarily, the culture media were removed to stop the internalization process and washed with ice-cold phosphate saline buffer (PBS) or serum-free medium followed by 2 × 5 min. The cells were incubated with 0.5 mL ice-cold acid wash buffer (0.02 M NaOAc, pH = 5) twice for 5 min to determine the cell-surface-bound (acid-releasable) radiotracer. Finally, 0.5 mL NaOH 1M was added to detach and lyse the 4T1 cells at 37 °C (three times). The internalized radioactivity was measured in a gamma counter, using the below formula (Equation (1)). The data for two fractions (cell surface-bound and internalized fraction) were collected in triplicate at the respective time points [55,56].

(1) $$Internalization(\%)=\frac{Internalized\;fraction}{surface\;bound\;fraction+Internalized\;fraction}\times 100$$

### 2.9. In Vitro Competitive Binding Study

A competitive binding assay was conducted to determine the value of 50% inhibitory concentration (IC_50_) for the [^68^Ga]Ga-DOTA-Ahx-peptide, using the non-radiolabeled peptide as a competitor. Approximately 1 × 10^5^ 4T1 cells per well in 24-well plates were maintained at 37 °C in a 5% CO_2_-humidified incubator for 2 days. On the day of the experiment, 45 nM of radiopeptide and a range of concentrations (10^−5^–10^−12^ M) of non-radiolabeled counterparts were incubated for 60 min. Subsequently, the supernatant was aspirated, and the cells were washed twice with ice-cold PBS. To determine the cell-associated radioactivity, cells were lysed by Triton X (0.5%) and 0.1 M NaOH. Thus, the radioactivity was measured using a gamma counter (RAYTEST, Straubenhardt, Germany) and the results were evaluated as the total binding (%) of radiopeptide in contrast to the logarithm (log) of the competition agent (M). The data were analyzed in GraphPad prism, and IC_50_ was calculated for the [^68^Ga]Ga-DOTA-Ahx-peptide [57].

### 2.10. Biodistribution Studies and Analysis

The biodistribution of [^68^Ga]Ga-DOTA-Ahx-peptide was evaluated in normal and tumor-bearing BALB/c mice. Therefore, 840 kBq in 100 μL saline was injected via the caudal vein of the mice. The animal was euthanized at different time points of 15, 30, 60, and 90 min post-injection using ketamine and xylazine in lethal doses. First, the blood sample was taken from the heart, and then organs (heart, kidney, liver, lungs, etc.) were removed. Tissues were rinsed and weighed, and the radioactivity of each organ was measured using a gamma counter. The organ uptake can be reported as the percentage of injected dose per gram of tissue (ID%/g). A 500-fold excess of DOTA-Ahx-ECRPPDDGLC was used 30 min before radiolabeled peptide injection for the blocking experiment [58,59].

### 2.11. PET/CT Imaging

Whole-body images were acquired on a PET/CT scanner system (Biograph 6, Siemens Medical Solutions, Erlangen, Germany). BALB/c mice received 5.5–7.4 MBq/100 μL of [^68^Ga]Ga-labeled peptide via the tail vein. After 30 and 60 min post-injection, mice were anesthetized by ketamine/xylazine and were placed in a prone position [60]. The CT scans were also performed for attenuation correction and anatomical imaging (20 s, 80 kV, 150mAs, and spatial resolution 1.25). Finally, PET images were reconstituted by a filtered back projection algorithm and fused with CT images. For binding specificity assessment, the unlabeled peptide was subcutaneously administrated at 30 min before the injection of the radiotracer [59].

## 3. Results

### 3.1. Synthesis and ^68^Ga-Labeling of the Peptide

**Figure 1:**
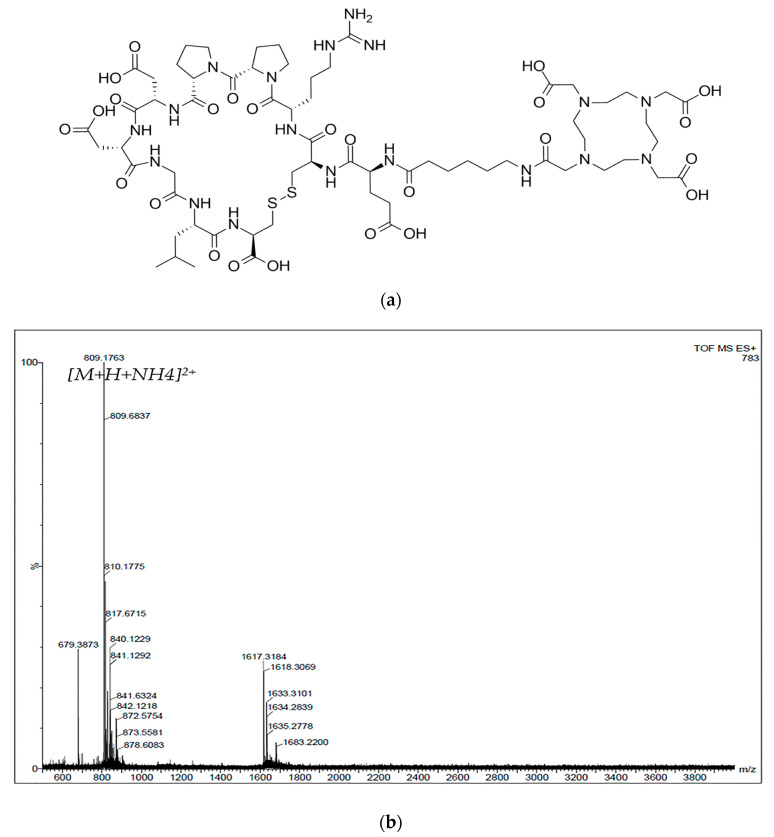
(**a**) The chemical structure of the DOTA-Ahx-peptide. (**b**) Mass spectrum for the DOTA-Ahx-peptide.

The designed peptide, DOTA-Ahx-VGB3 (Figure 1a), was synthesized manually on the 2-chlorotrityl chloride resin, using SPPS. Some organic compounds such as TBTU (2-(1H-benzotriazole-1-yl)-1,1,3,3-tetramethylaminium tetrafluoroborate), DIPEA (N,N-diisopropylethylamine), and DMF (dimethylformamide) were used for reaction media, forming a peptide bond. The final product was prepared as a white powder and purified with HPLC (>86%). LC-MS analysis was performed for the synthesized structure with a molecular weight of 1600.68 g/mol found; *m*/*z* = 809.5 g/mol [M+H+NH_4_]^2+^ (Figure 1b).

**Figure 2:**
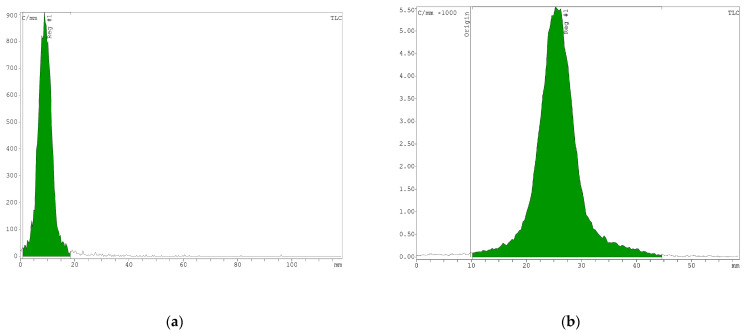

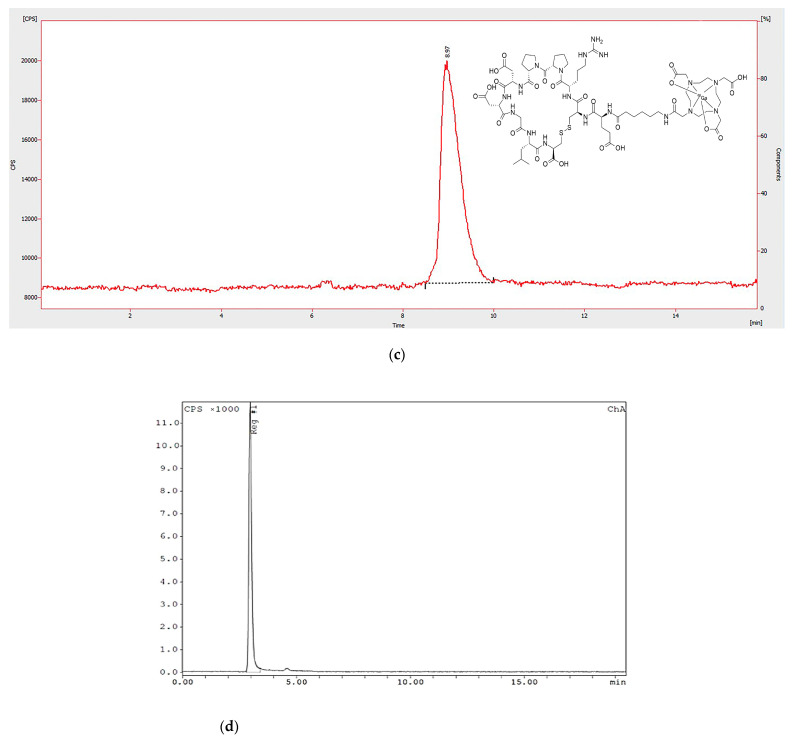
Radiochemical purity analysis of ^68^GaCl_3_ and [^68^Ga]Ga-DOTA-Ahx-VGB3. ITLC results of (**a**) ^68^GaCl_3_ and (**b**) [^68^Ga]Ga-DOTA-Ahx-VGB3. HPLC profiles of (**c**) [^68^Ga]Ga-DOTA-Ahx-peptide and (**d**) ^68^GaCl_3_, using the C_18_ column. The peaks at 3.02 and 8.97 min are related to ^68^Ga free and [^68^Ga]Ga-DOTA-Ahx-peptide, respectively.

The radiolabeling of the DOTA-Ahx-peptide was generated with a high radiochemical yield (>95%). The radiochemical purity was 98% and was evaluated by ITLC. For comparison, an aliquot of ^68^GaCl_3_ solution from the generator was spotted on ITLC-SG and analyzed by a radio-TLC scanner. RP-HPLC was also performed to confirm the absence of radiochemical impurities and radiolysis. For more investigation, an aliquot of ^68^GaCl_3_, eluted from the generator, was also injected into the HPLC. As displayed in Figure 2, the retention times of ^68^Ga free and [^68^Ga]Ga-DOTA-Ahx-peptide on RP-HPLC were at 3.02 and 8.97 min, respectively.

### 3.2. n-Octanol/Water Partition Coefficient (Log p)

The lipid–water partition coefficient for the [^68^Ga]Ga-DOTA-Ahx-peptide was estimated in the n-octanol water system, using a gamma counter. Approximately, the log p value was −2.17 ± 0.072, considering the below formula (Equation (2)). The result is reported as mean ± SD (*n* = 3). The calculated log p indicates that this radioligand is a very hydrophilic agent.

(2) $$\log p=\frac{Activity\;in\;n\_octanol\;}{Activity\;in\;aqueous\;phase}$$

### 3.3. In Vitro Stability of the Radiopeptide

**Figure 3:**
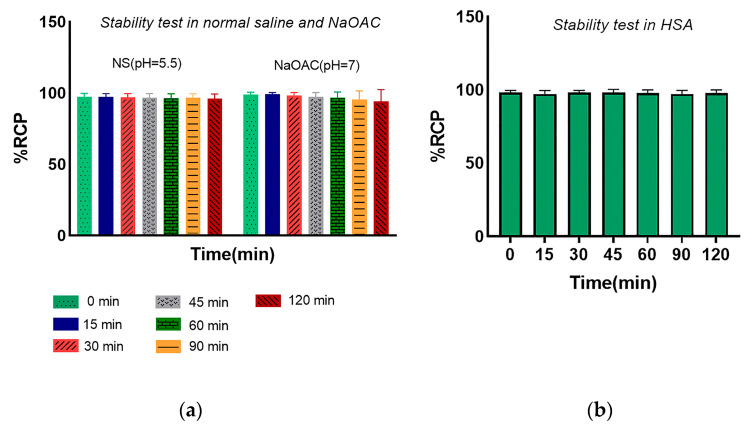
Stability test of the [^68^Ga]Ga-DOTA-Ahx-peptide in (**a**) normal saline and 1 M sodium acetate and (**b**) human serum albumin.

The stability test was evaluated for the radiolabeled peptide under different conditions (pH = 5.5 and 37 °C) in normal saline (pH = 7, 1 M) and sodium acetate buffer and in HSA for 120 min. The percentage of intact radiopeptide was analyzed by radio-TLC at various time points in methanol/ammonium acetate 1:1 (*V/V*). Thus, the radiolabeled peptide showed more than 95% stability in normal saline and 1 M sodium acetate buffer (Figure 3). Post-incubation of the radiotracer in human serum albumin at different time intervals demonstrated high stability, remaining >95% even after 120 min.

### 3.4. Radionuclide Identification and Activity Measurements

**Figure 4:**
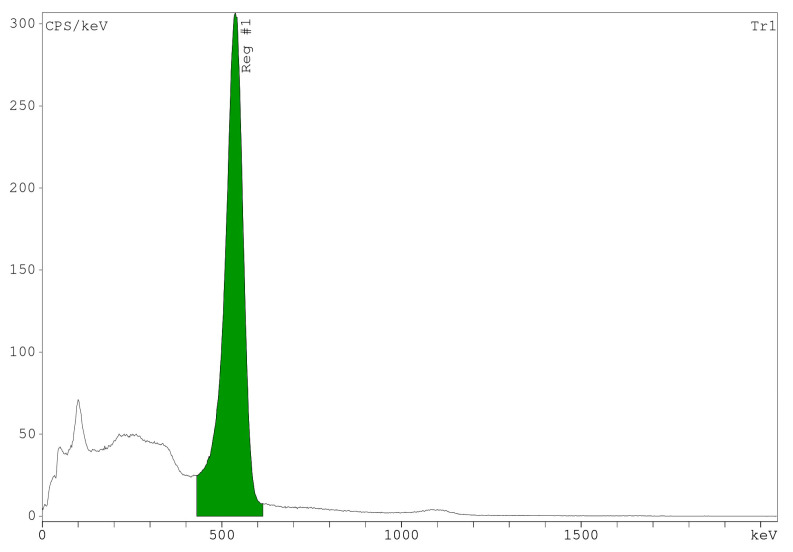
The result of gamma spectrometry for ^68^Ga identification.

Gamma spectrometry was performed based on the main emitted gamma from the radionuclide. The spectrum showed a peak at the energy of 0.511 MeV, relating to the main peak of ^68^Ga (Figure 4). Additionally, the half-life was calculated with the mentioned formula and confirmed the physical half-life of ^68^Ga, which was about 67.41 ± 1.03 min.

### 3.5. Internalization and Surface Binding in the Breast Cancer Cell Line

**Figure 5:**
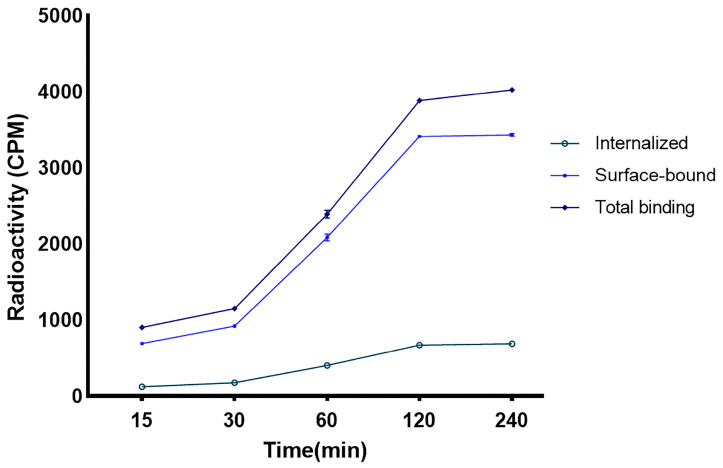
Internalized and surface-bound assay of the [^68^Ga]Ga-DOTA-Ahx-peptide.

For the internalization experiment, an overexpressed VEGFR-1/2 cell line was plated at a density of 10^6^/well and allowed to incubate with constant concentrations of radioligand. Based on the bound and internalized radioactivity, the radiolabeled peptide showed binding to VEGFR1/2. The binding of the [^68^Ga]Ga-DOTA-Ahx-peptide in 4T1 cells increased over 120 min and then plateaued at the last time interval. As indicated in Figure 5, approximately 17% of the total uptake was internalized after 2 h incubation, and 83% was related to a surface-bound fraction.

### 3.6. Competitive Cell Binding

**Figure 6:**
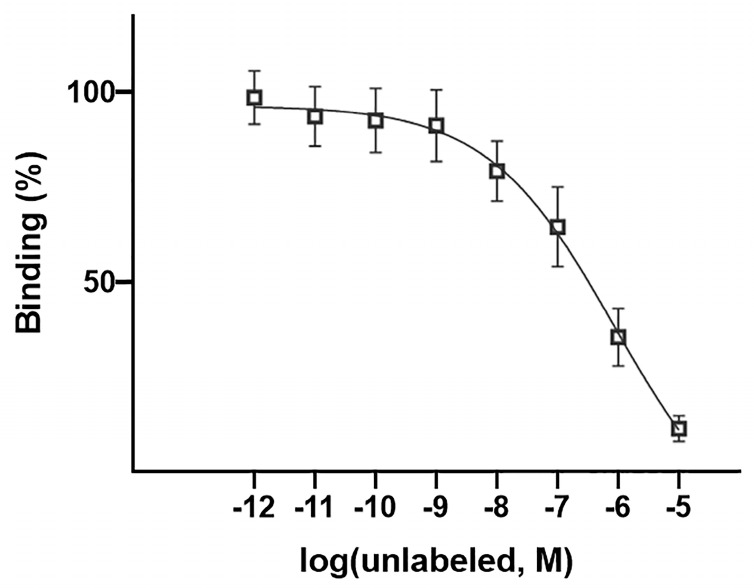
Competitive binding assay of the [^68^Ga]Ga-DOTA-Ahx-peptide using 4T1 cells.

Binding affinity was investigated by a competitive cell-binding study using increasing concentrations of unlabeled peptide. The data showed a decrease in the 4T1-bound radioactivity implication of the radioligand displacement. As demonstrated in Figure 6, the IC_50_ value for the [^68^Ga]Ga-DOTA-Ahx-peptide was about 867 nM.

### 3.7. Ex Vivo Biodistribution Studies

**Figure 7:**
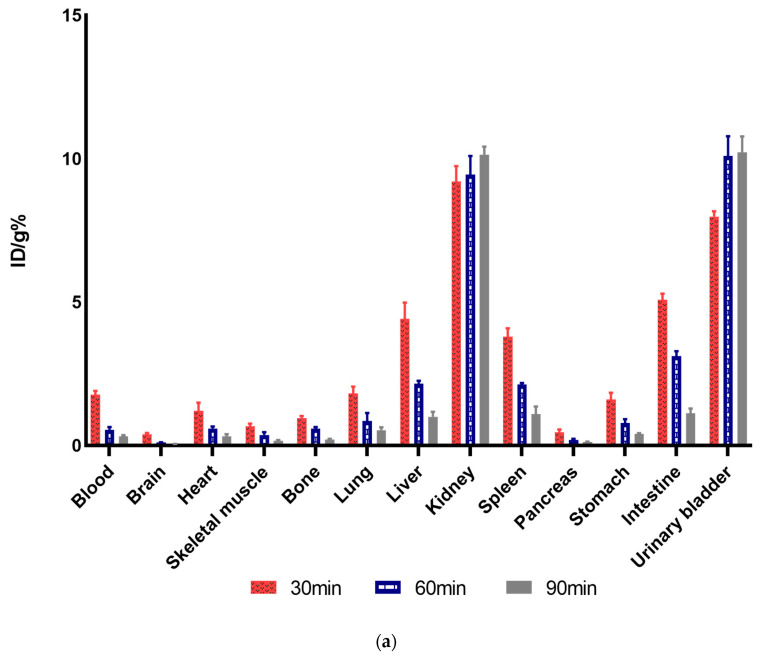

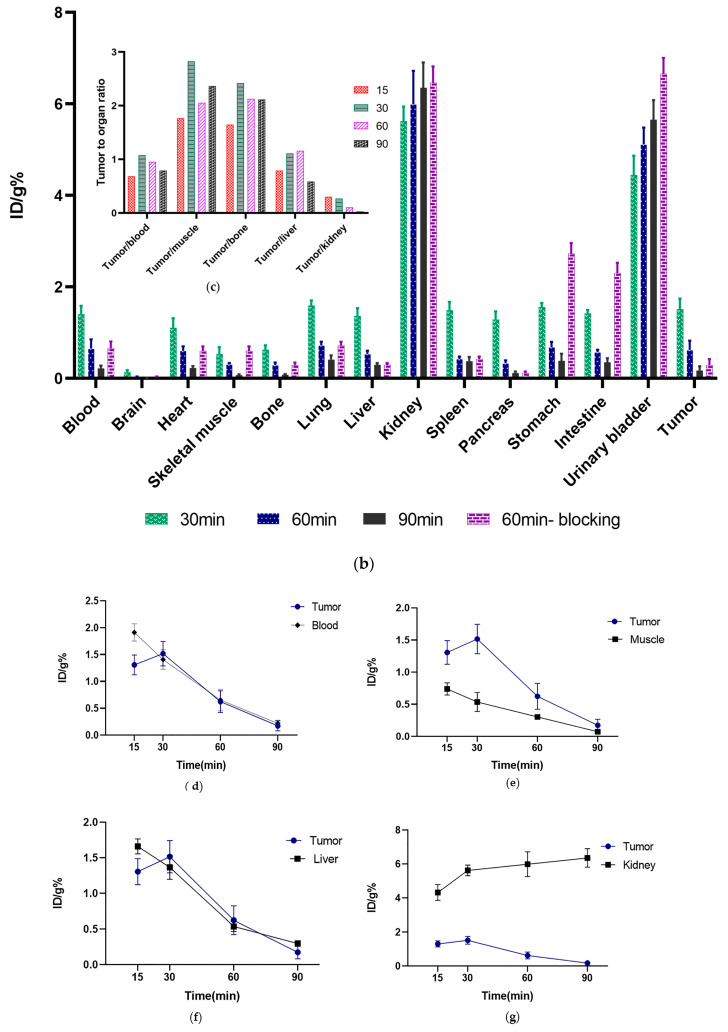
The biodistribution of the [^68^Ga]Ga-DOTA-Ahx-peptide in (**a**) normal and (**b**) tumor-bearing BALB/c mice. The data are described as %ID/g, calculated at 30, 60, and 90 min after injection. The results are reported as mean ± SD (*n* = 3). (**c**) Tumor-to-organ ratio at 30, 60, and 90 min after IV injection. The uptake profile of (**d**) blood, (**e**) muscle, (**f**) liver, and (**g**) kidneys in comparison with the tumor from 15 to 90 min.

Comparative biodistribution studies were conducted in healthy (Figure 7a) and subcutaneously (s.c) tumor-bearing mice (Figure 7b). As displayed, [^68^Ga]Ga-DOTA-Ahx-VGB3 in blood decreased rapidly from 1.7. ± 0.13%ID/g to 0.32 ± 0.036%ID/g for healthy mice and from 1.4 ± 0.18%ID/g to 0.22 ± 0.06%ID/g for tumor-bearing mice at 30 and 90 min post-injection (p.i.). The same kinetics profile was observed in highly vascularized organs, including the heart, lung, and liver. In addition, the maximum uptake of the liver was found to be 4.41 ± 0.56%ID/g in normal mice at 30 p.i. Relatively high uptake was also observed in healthy tissues such as the spleen and intestine (3.7 ± 0.29%ID/g and 5.07 ± 0.21%ID/g, respectively). At 30 min, the highest accumulation of the radioactive compound was measured in the kidneys, which was 9.2 ± 0.53%ID/g and increased over time up to 90 min. Considering kidney uptake, this tracer is excreted mainly from this organ. The distribution profile of the [^68^Ga]Ga-DOTA-Ahx-peptide in tumor-bearing mice had some similarity to the normal mice. Kidneys in tumor-bearing mice were also the critical organ with the highest radioactivity concentration at different time points (5.62 ± 0.31%ID/g at 30 min and 6.35 ± 0.54%ID/g at 90 min). This increasing profile has confirmed the quick washing-out of important organs such as the heart, lungs, liver, stomach, and intestine, which had relatively high radioactivity uptake. The blocking study was also conducted and revealed a decrease of more than 50% in tumor uptake at 60 min after injection. As shown in Figure 7c, the maximum ratio of tumor to blood %ID/g(T/B) was 1.07 at 30 min, which decreased to 0.78 at 90 min. The ratios of the other organs, including tumor to the muscle (T/M = 2.82 at 30 min postinjection), tumor to the liver, etc., are also demonstrated in this figure. Additionally, Figure 7d–g displays the uptake profile of the blood, muscle, liver, and kidneys compared to tumor uptake. The maximum tumor uptake was 30 min after injection (1.51 ± 0.22%ID/g) and decreased by 90 min.

### 3.8. PET-CT Imaging

**Figure 8:**
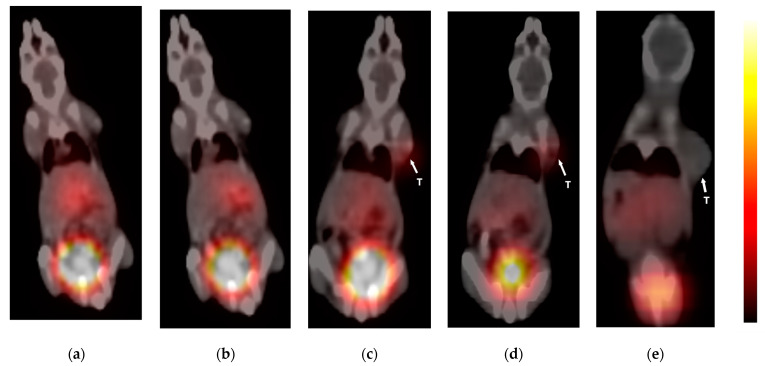
PET/CT imaging of the [^68^Ga]Ga-DOTA-Ahx-peptide in normal mice at (**a**) 30 min and (**b**) 60 min after IV injection, and tumor-bearing BALB/c mice at (**c**) 30 min and (**d**) 60 min after IV injection; (**e**) 60 min blocking using the DOTA-Ahx-peptide. White arrowhead indicates tumor (T = tumor).

The acquisitions through the [^68^Ga]Ga-DOTA-Ahx-peptide were obtained in healthy and 4T1 tumor-bearing BALB/c mice, using PET fused with CT that allows for a visual and direct assessment of a radiotracer. Figure 8 shows the PET/CT scan of the [^68^Ga]Ga-DOTA-Ahx-peptide in normal and tumor-bearing mice at 30 and 60 min after injection. The images show that the [^68^Ga]Ga-DOTA-Ahx-peptide can accumulate in the tumor site. Both healthy and tumor-bearing mice exhibited remarkable radiotracer accumulation in the kidneys and urinary bladder, indicating that renal excretion is an important elimination route. Nevertheless, this was expected according to the hydrophilic property of the [^68^Ga]Ga-DOTA-Ahx-peptide. The PET scans at 30 and 60 min also confirmed the biodistribution experiments, which demonstrated a rapid clearance from vascularized organs including the heart, lung, and liver over time. As displayed, the uptake of these organs decreased in the interval of 30 to 60 min. On the other hand, the tumor uptake of the [^68^Ga]Ga-DOTA-Ahx-peptide was significantly reduced by blocking agent, using an unlabeled counterpart.

All supporting information and additional data are available at Supplementary Materials.

## 4. Discussion

As VEGFR-1/2 has a high expression on tumor cells and correlates to angiogenesis and tumor progression, it can be considered as an excellent target for the early detection of breast cancer [61,62,63]. In the present study, VGB3, a VEGFR1/2-targeting peptide, was selected as the promising agent for VEGFR-1/2-positive tumor detection. This peptide could inhibit angiogenesis and proliferation via VEGFR-1/2 binding and showed tumor volume regression in the 4T1 mice model, representing the antitumor activity of VGB3 [64,65].

An Ahx-linker was added between the peptide core and DOTA-chelator to improve tumor targeting and binding affinity. Thus, the DOTA-Ahx-peptide was synthesized by SPPS and radiolabeled with ^68^Ga in order to assess the feasibility [^68^Ga]Ga-DOTA-Ahx-VGB3 for PET imaging of VEGFR-1/2 by in vivo and in vitro experiments. Generally, PET imaging through ^68^Ga can provide a high spatial resolution and sensitivity compared to SPECT imaging, leading to better diagnosis [66].

[^68^Ga]Ga-DOTA-Ahx-VGB3 was prepared with high radiochemical purity (98%), confirmed by chromatographic methods [49]. The log P was estimated at −2.17 ± 0.072, which indicates the highly hydrophilic property of radiotracer and predominantly renal route of excretion. [^68^Ga]Ga-DOTA-Ahx-VGB3 showed high in vitro stability in different buffer systems, determined by ITLC (>95%). Therefore, even after 120 min post-incubation, the radiolabeled peptide remained stable.

The in vitro competition assay demonstrated the specificity of [^68^Ga]Ga-DOTA-Ahx-VGB3 for 4T1 cancer cells, implying suitable affinity to VEGFR1/2. Approximately 17% of the radiotracer was internalized after 2 h incubation, and 83% of binding was related to a surface-bound fraction. Thus, the majority of radiopeptide binding occurred on the cell surface. On the other hand, the internalized fraction can be favorable for imaging studies and may contribute to prolonged retention in tumor tissue [67].

The [^68^Ga]Ga-DOTA-Ahx-peptide was also evaluated as an imaging agent by PET-CT in healthy and 4T1 tumor-bearing BALB/c mice and ex vivo biodistribution at 30 and 60 min after injection. Ex vivo biodistribution studies for the [^68^Ga]Ga-DOTA-Ahx-peptide exhibited relatively low uptake in the organs, except for the kidneys. Indeed, both healthy and tumor-bearing mice showed remarkable uptake in the kidneys and urinary bladder, which represents renal excretion as a main elimination route. These results follow previously reported data for VEGFR-targeting peptides [30,33].

The tumor uptake 30 min p.i. was 1.51 ± 0.22%ID/g, and compared with the research of Rezazadeh et al., we obtained very similar results [68]. The accumulation of radiopeptide in tumors seems to be rapid. This issue is indicated by the maximum tumor uptake of the [^68^Ga]Ga-DOTA-Ahx-peptide at 30 min post-injection. Tumor-to-blood and tumor-to-muscle ratios are important ex vivo factors to predict suitable tumor-to-background contrast, resulting in better images. In this respect, the [^68^Ga]Ga-DOTA-Ahx-peptide showed tumor-to-blood and tumor-to-muscle of 1.07 and 2.82 at 30 min p.i., respectively. These findings are in agreement with previous studies, where the tumor-to-blood ratio was <2 and tumor uptake was 2–6-fold higher than muscle [29,30,68]. Additionally, the ratio of tumor to blood suggests that due to peptide binding affinity toward VEGFR-1/2, the blood clearance was slightly higher than the tumor, which can result in suitable tumor uptake. Finally, the blocking study was also conducted and revealed a decrease of more than 50% in tumor uptake of radiopeptide 60 min after injection, referring to the suitable specificity of radiopeptides.

## 5. Conclusions

The [^68^Ga]Ga-DOTA-Ahx-VGB3 was successfully synthesized in a high yield and radiochemical purity. Preclinical studies have shown that [^68^Ga]Ga-DOTA-Ahx-VGB3 specifically targets VEGFR-1/2, allowing an appropriate tumor accumulation (2–3-fold higher than muscle). This was confirmed by the blocking study, using the unlabeled peptide, which showed more than a 50% reduction in tumor uptake. Thus, [^68^Ga]Ga-DOTA-Ahx-VGB3 might be a promising molecular probe for the noninvasive visualization of VEGFR-1/2 expression, which provides a basis for several investigations using further modifications on the linker, chelator, and even the peptide core.

## Data Availability

Data are available from the corresponding author on reasonable request.

## Supplementary Materials

The following supporting information can be downloaded at https://www.mdpi.com/article/10.3390/pharmaceutics16070899/s1. Figure S1: Mass spectrum of DOTA-Ahx-Linear peptide before disulfide bond formation. Figure S2: HPLC result for the DOTA-Ahx-Linear peptide. Figure S3: HPLC result for the DOTA-Ahx-VGB3 (after purification). Figure S4: PET/CT imaging of [^18^F]F-FDG in tumor-bearing BALB/c mice at 60 min after IV injection for the validation of tumor modeling.
