# Supplementary material for: Synthesis, Radiolabeling, and Biodistribution Study of a Novel DOTA-Peptide for Targeting Vascular Endothelial Growth Factor Receptors in the Molecular Imaging of Breast Cancer
Source: Pharmaceutics. 2024 Jul 4;16(7):899. doi: 10.3390/pharmaceutics16070899 (PMC11279866; doi:10.3390/pharmaceutics16070899)
Supplement: Supplementary file 1 [file pharmaceutics-16-00899-s001.zip › pharmaceutics-2753339-supplementary.pdf]

Table of content

Figure S1: Mass spectrum of DOTA-Ahx-Linear peptide before disulfide bond formation.

Figure S2: HPLC result for DOTA-Ahx-Linear peptide.

Figure S3: HPLC result for DOTA-Ahx-VGB3(after purification).

Figure S4: PET/CT imaging of [^{18}F]F-FDG in tumor-bearing BALB/c mice at 60 min after IV injection for validation of tumor modeling.

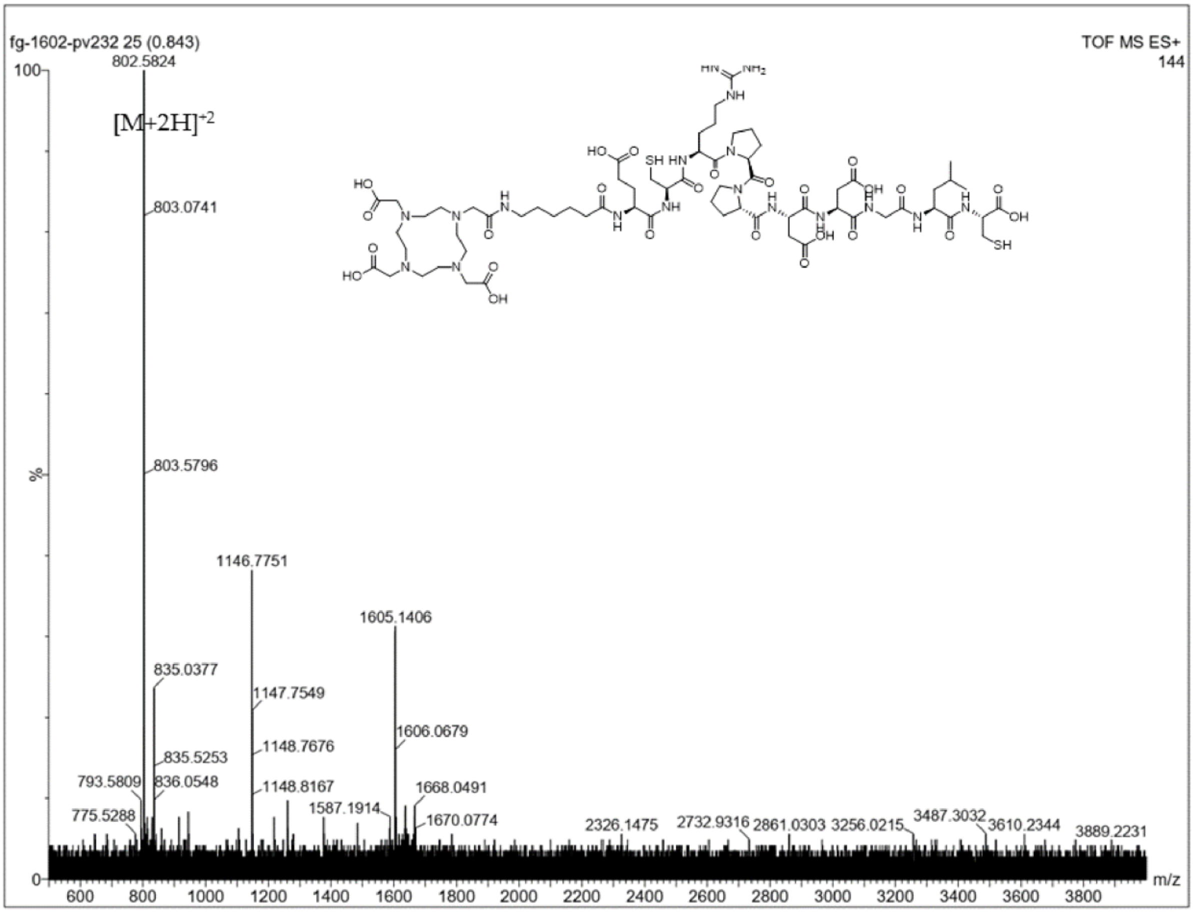

Figure S1: Mass spectrum of DOTA-Ahx-Linear peptide before disulfide bond formation (MW= 1602.70).

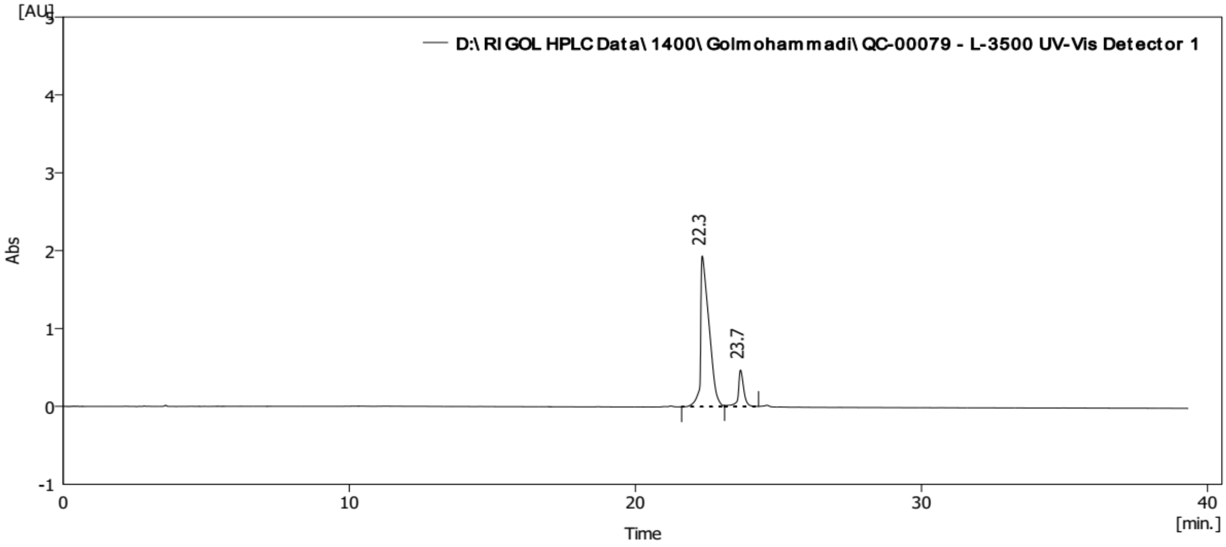

Result Table (Uncal - D:\RIGOL HPLC Data\1400\Golmohammadi\QC-00079 - L-3500 UV-Vis Detector 1)

	Reten. Time [min]	Area [mAU.s]	Height [mAU]	Area [%]	Height [%]	W05 [min]	Compound Name
1	22.337	39811.785	1936.505	86.6	80.5	0.32	
2	23.677	6160.462	468.663	13.4	19.5	0.18	
	Total	45972.247	2405.168	100.0	100.0		

Figure S2: HPLC result for DOTA-Ahx-Linear peptide.

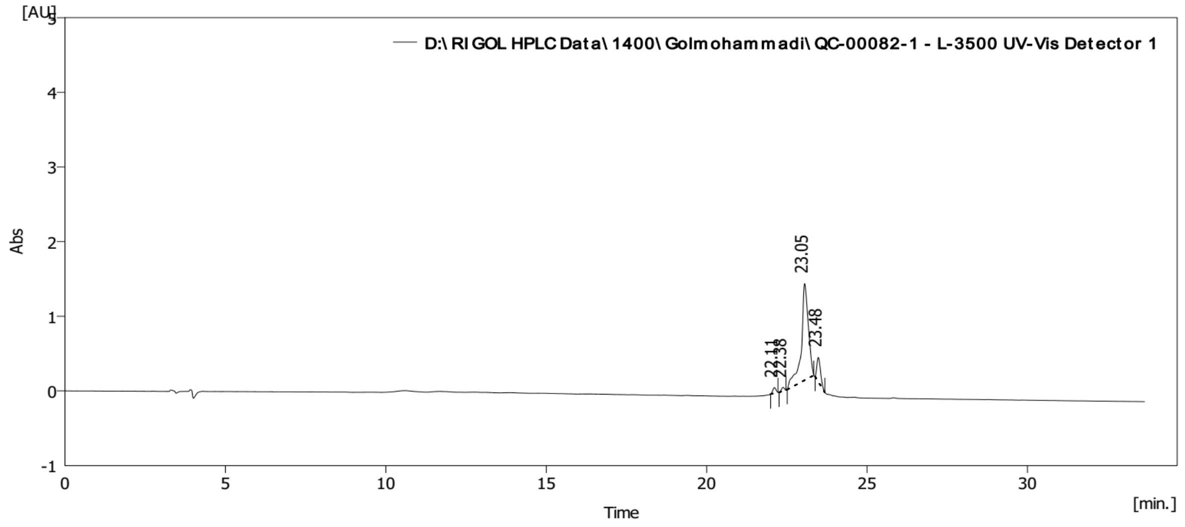

Result Table (Uncal - D: \RIGOL HPLC Data\1400\Golmohammadi\QC-00082-1 - L-3500 UV-Vis Detector 1)

	Reten. Time [min]	Area [mAU.s]	Height [mAU]	Area [%]	Height [%]	W05 [min]	Compound Name
1	22.113	510.808	75.160	2.2	4.3	0.12	
2	22.380	322.312	49.742	1.4	2.8	0.11	
3	23.053	20019.796	1295.053	85.3	74.2	0.20	
4	23.480	2608.508	325.614	11.1	18.7	0.12	
	Total	23461.424	1745.569	100.0	100.0		

Figure S3: HPLC result for DOTA-Ahx-VGB3 (after purification).

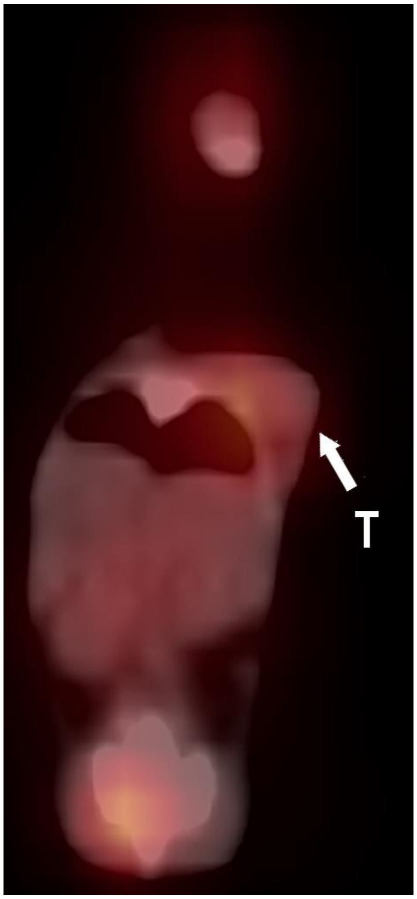

Figure S4: PET/CT imaging of [^{18}F]F-FDG in tumor-bearing BALB/c mice at 60 min after IV injection for validation of tumor modeling (T=Tumor)
